# Establishing data governance for sharing and access to real-world data: a case study

**DOI:** 10.1093/jamiaopen/ooaf041

**Published:** 2025-06-23

**Authors:** Heath A Davis, Diva Kerkman, Asher A Hoberg, Michele Countryman, Wendy Beaver, Kiley Bybee, James M Blum, Boyd M Knosp

**Affiliations:** Office of Information Technology, Roy J. and Lucille A. Carver College of Medicine, University of Iowa, 200 Newton Road, Iowa City, IA 52242, United States; Biomedical Informatics, Institute for Clinical and Translational Science, University of Iowa, 200 Hawkins Drive, Iowa City, IA 52242, United States; Biomedical Informatics, Institute for Clinical and Translational Science, University of Iowa, 200 Hawkins Drive, Iowa City, IA 52242, United States; Office of Information Technology, Roy J. and Lucille A. Carver College of Medicine, University of Iowa, 200 Newton Road, Iowa City, IA 52242, United States; Biomedical Informatics, Institute for Clinical and Translational Science, University of Iowa, 200 Hawkins Drive, Iowa City, IA 52242, United States; Human Subjects Office, Office of the Vice President for Research, University of Iowa, 600 Newton Rd #105, Iowa City, IA 52245, United States; Division of Sponsored Programs, Office of the Vice President for Research, University of Iowa, 100 Gilmore Hall 112 N. Capitol St, Iowa City, IA 52242, United States; Joint Office for Compliance, Iowa Health Care, University of Iowa, 200 Hawkins Drive, Iowa City, IA 52242, United States; Health Care Information Systems, Iowa Health Care, University of Iowa, 200 Hawkins Drive, Iowa City, IA 52245, United States; Department of Anesthesia, Iowa Health Care, University of Iowa, 200 Hawkins Drive, Iowa City, IA 52245, United States; Office of Information Technology, Roy J. and Lucille A. Carver College of Medicine, University of Iowa, 200 Newton Road, Iowa City, IA 52242, United States; Biomedical Informatics, Institute for Clinical and Translational Science, University of Iowa, 200 Hawkins Drive, Iowa City, IA 52242, United States

**Keywords:** data governance, real-world data, clinical research data, Enterprise Data Warehouse for Research, external data sharing for research, research data

## Abstract

**Importance:**

Data governance, the policies, and procedures for managing data, is a critical factor for secondary use of clinical data for research.

**Objectives:**

This paper describes the evolution of an academic health-care organization’s data governance for research, development of an external data sharing process, implementation of related processes, continuous improvement, and ongoing observations of data governance maturity.

**Materials and Methods:**

The program was designed to improve the access to and sharing of real-world data for research. Using a combination of qualitative and quantitative methods, we evaluated the program’s effectiveness.

**Results:**

Our results describe a significant improvement in data accessibility as seen in new data-driven performance indicators and in data understanding indicated by new processes, policies, and strategies.

**Discussion:**

The paper outlines the development of a data governance process at an academic health center to support external data sharing, emphasizing the importance of data literacy, cross-office collaboration, and structured workflows to manage complex review requirements. The formalized process improved data access, identified gaps, and enabled continuous quality improvement, though it introduced new bottlenecks and required navigating multi-office reviews and researcher education.

**Conclusion:**

These findings suggest data governance practices that may apply to other institutions.

## Objective

Clinical data governance is an organizational practice to ensure health systems are leveraging data appropriately for data-driven decision-making and improving patient outcomes. Data governance involves defining policies and procedures for managing clinical data to ensure its accuracy, completeness, consistency, and security.^1^ These policies and procedures include standards for data collection, storage and sharing, roles and responsibilities for managing data throughout its lifecycle, and the management and implementation of these decisions. Data governance also enables efficient secondary use of clinical data for research.

At academic medical centers, data governance is critical for determining access to and sharing of real-world data as part of clinical care (eg, medical documentation, employee/staffing data, billing, etc.) such as electronic health records (EHRs).^2^ Effective data governance optimizes and expedites the use of data accelerating clinical and translational science.

In the context of the health-care covered entity (HCE), formal, documented, and reproducible processes had not been established to address research-based requests for sharing data outside of the HCE—including sharing data with research units on campus and external organizations—resulting in ad hoc processes that caused delays, missed opportunities, and a gap in fully utilizing data for discovery. Clinical research data governance development stemmed from one of the goals of the Iowa Health Data Resource (IHDR) to increase access and literacy to clinical data for research on campus.^3^ The IHDR was based on Maturity Model^4^ research on Enterprise Data Warehouses for Research (EDW4R).^5^ A maturity model is a framework that helps organizations assess and improve their processes, capabilities, and performance that have been used extensively in information technology. Models typically consist of several levels or stages, each representing a different degree of maturity. As organizations progress through these levels, they implement more sophisticated practices and achieve higher quality outcomes. The EDW4R Maturity Model examines: (1) access and outreach: strategies for engaging researchers and facilitating their access; (2) service management: the degree of formalization in service offerings; (3) workforce: characteristics and composition of the EDW4R team; (4) relationship to enterprise IT: the level of engagement between the EDW4R team and central IT units; (5) data governance: decision-making processes regarding EDW4R; and (6) metrics: methods for measuring EDW4R operations. From this, based on our self-analysis of maturity level, our organization identified 3 areas of opportunity, and developed a strategic project to address these opportunities with the following goals: (1) increasing literacy and access to clinical data for research; (2) establishing a data enclave for the use of data out of the EHR; and (3) the establishing transformative datasets for research.^3^ This allowed us to address specific gaps identified and leverage learnings from peer organization who had were more mature in these areas. It became apparent during the development and implementation of the IHDR that there were gaps in our clinical data governance for research processes.

This paper details an approach to clinical data governance for research and outlines the organizational structure, the leadership charge, processes, and tools used to enable effective access and sharing of real-world data, including: the evolution of University of Iowa Health Care’s (UIHC’s) data governance for research; the development of a formal external data sharing workflow; the implementation of related processes and continuous process improvement; and ongoing observations of data governance maturity.

## Methods

A Donabedian structure, process, outcome approach was taken to guide this pilot.^6^^,^^7^ Preparation activities included highlighting the problem, engaging leadership, and establishing a working group. Process review and refinement included identifying key elements, evaluating communication and literacy, and defining needed workflows.

### Preparation

#### Highlighting the problem

Through the establishment of the IHDR data ecosystem, health science researchers across the organization had greater literacy and access to clinical data for research. This resulted in increasing demand for data and data sharing and highlighted the need for formal external data sharing processes for research. As the volume of data sharing requests increased, it became clear that there were gaps and inconsistencies in the process.

#### Leadership engagement

Engaging leadership was critical to insure buy-in and cooperation across the various entities involved in data sharing requests. The UIHC Data Governance Task Force (Task Force) was created and charged with the responsibility of managing the organization’s information and data-related objectives. The Task Force ensures alignment with the overall organizational goals^8^ by leveraging existing review structures in place to follow federal data sharing and public access policies within the Division of Sponsored Programs (DSPs) and Iowa’s Human Subject Office, Institutional Review Board (HSO-IRB). Specifically, the Task Force proposes recommendations to enhance data quality, strike a balance between data access and security, prioritize data acquisition and distribution efforts, and promote data literacy across campus.

#### External Data Sharing Working Group

To better understand these requests to share data for research purposes and address the increased demand, the Task Force established and empowered an External Data Sharing Working Group (Working Group) consisting of people with strategic roles across the organization^9^ to review and make recommendations regarding data sharing. The Working Group—consisting of experts from the Joint Office of Compliance, HSO-IRB, DSP, Information Security and Policy Office, Enterprise Clinical Data Analytics, and Biomedical Informatics—was charged with reviewing requests to share data and make recommendations to the Task Force.

### Processes

#### Communication and literacy

As this initiative began, there were numerous questions from the offices involved in the working group and the research community. There was also some resistance to the nascent processes. The questions and resistance made it clear there was a lack of shared understanding as to when and why an external data sharing request was required. Two tools were developed to improve communication for both the research community and offices with oversite of various parts of the data sharing process. A preliminary decision workflow diagram (Figure S1) and a simple self-service data governance recommender tool (Figure S2). It was determined that this pilot’s outcome would be a model and process to facilitate the documentation, review, and execution of the data sharing requests in a unified manner for the Working Group and Task Force.

#### Key elements

The first activity was identifying key elements about each data sharing request that needed to be captured. The Task Force and Working Group identified key elements starting with 28 free-text questions such as, who is the Principal Investigator, with whom will the data be shared, and who owns any intellectual property developed. The Working Group reviewed the initial list, suggested additional information needed to fully understand data sharing requests and recommended categorization of variables to facilitate a structured decision-making process. The resultant key elements consist of 47 structured and free-text questions (Appendix S1) used to develop the pilot external data sharing request workflow.

#### Pilot

**Figure 1.**
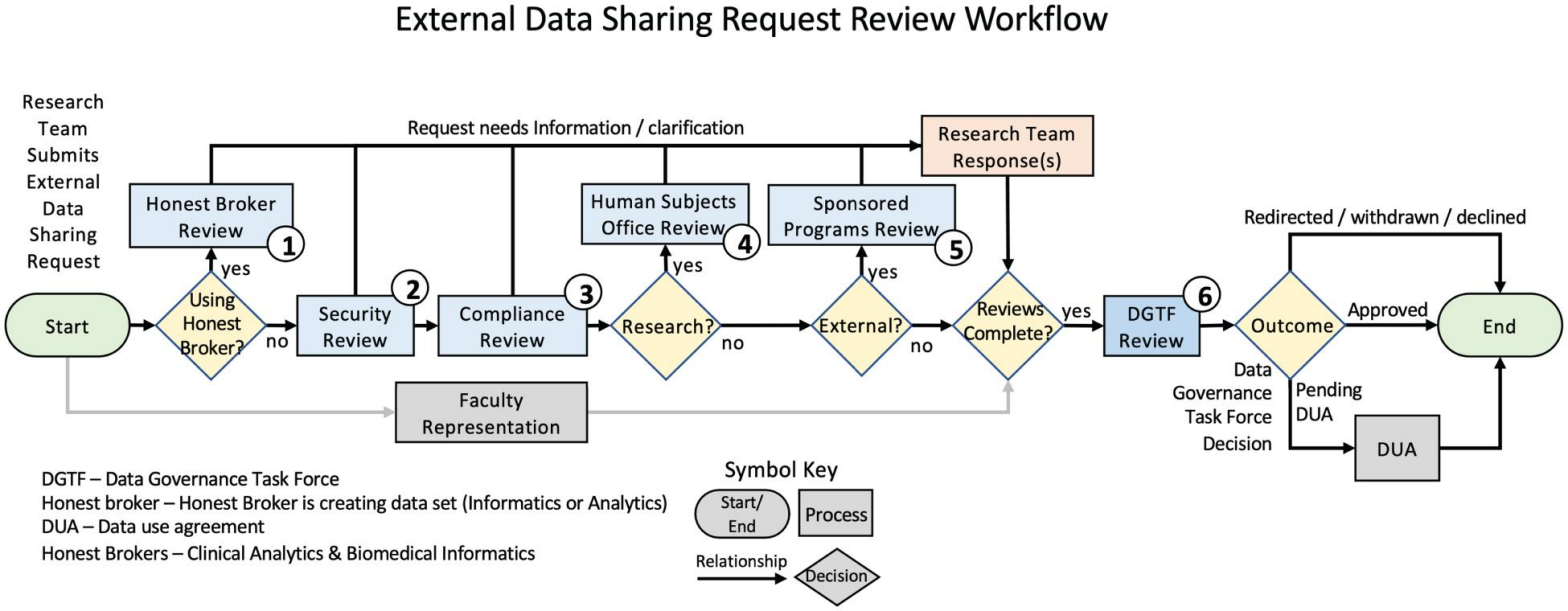
At stops 1-5, the outcomes can be (1) request for information or clarification from the researcher, (2) reviewed and send to the next stop without concerns, or (3) reviewed and send to the next stop with concerns documented. At stop 6, the request is approved with the Data Use Agreement Approved internal sharing with the Internal Data Use Agreement, redirected to existing resources within health care.

Using the key data elements, a draft of External Data Sharing Review Workflow Model was constructed in REDCap^10^ before finalization. The pilot workflow was used to test the working model and processes, and document feedback from the research community and the Working Group. Multiple iterations tested various external sharing use cases and built consensus among stakeholders, a key to effective governance^2^ and establishing formal processes. During the pilot, an informatics staff served as an External Data Sharing Navigator. They facilitated requests, communicated between stakeholders, assisted researchers with the request form questions from the Working Group, and documented feedback, gaps, and opportunities, that were worked into a cycle of process improvement prior to the final product. The pilot model evolved and was deployed into an enterprise-wide workflow solution. This workflow and documentation process will allow research teams to see which Working Group office is reviewing their request and to address and review comments directly from the Working Group. The enterprise deployment incorporated logical automation to streamline requests through the workflow while ensuring transparent communication and comments between offices (Figure 1).

## Results

The outcomes of this pilot include the development of a final External Data Sharing Request Determination model and an External Data Sharing Review Workflow Model. Additionally, the Working Group has been empowered to review and manage these requests in a consistent, cohesive manner, with trackable metrics.

### External Data Sharing Request Determination

**Figure 2.**
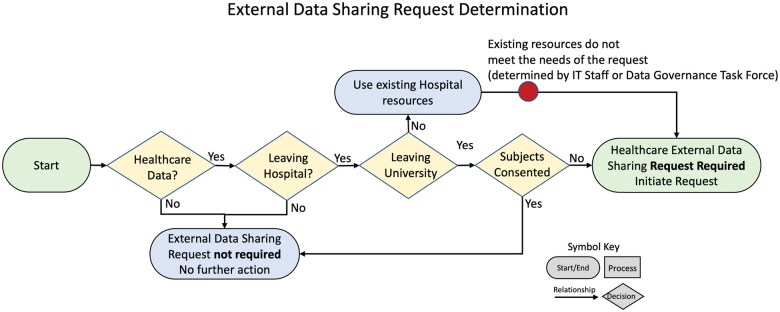
The process includes determining if: (1) the request contains health-care data, (2) data are leaving health care, (3) data are leaving the university, and (4) subjects are consented for data sharing.

The first step in the clinical data sharing request process is determining if the project meets with requirements of data sharing request. The workflow for External Data Sharing Determination (Figure 2) is as follows: for data that does not originate in the health system, a Sharing Request is not required, no further action is needed for the principal investigator (PI). For data that originate in the health system: if data are not leaving the HCE, a Sharing Request is not required, no further action is needed for the PI. If data leave the HCE and they are not leaving the University, the request is redirected to use resources within the HCE unless informatics staff has determined that the resources within HCE do not meet the requirements for analysis. If data are leaving the HCE, the University and subjects have consented to share data, a Sharing Request is not needed, no further action is needed for the PI. If data are leaving both the HCE, the University, and subjects are not consented for sharing data, a Sharing Request is required. The determination was used: to clarify which requests needed to go through a Sharing Request, which requests did not; to develop a straightforward way to communicate the new process with the research community; and to ensure that the stakeholders on the Working Group had shared understanding of the processes. This determination workflow is the minimum data footprint needed to establish if a Data Sharing was required. This process allows the organization to understand exactly how and why health-care data are being shared, with whom they are being shared, provides guidance and oversight to researchers who are unfamiliar with sharing organizational health data externally, and a documentation record of this determination.

### External Data Sharing Review Workflow Model

Based on the testing and refinement of the REDCap-based draft model described in the “Methods” section, the final workflow model, a “train track” approach was established with defined, ordered stops. The first stop in the process is with the honest broker groups (eg, Clinical Analytics or Biomedical Informatics Core). The honest broker team either reviews the request if the research study team indicated they are using an honest broker, or they mark not applicable. If an honest broker is not used to acquire the data, a higher level of scrutiny is applied during the process. The second and third mandatory stops are with the Information Security and Policy Office followed by the Office of Compliance. If the data sharing is related to research, the HSO-IRB is the fourth stop. For data leaving the university, Sponsored Programs is the last stop. In the pilot, this was typically the stop that took the longest to complete due to internal and external organization requirements for executing data sharing agreements. Once all the stops from the Working Group are complete, a recommendation is sent to the Task Force. At each stop, the Working Group member can: (1) request information or clarification from the researcher, (2) review and send to the next stop without concerns, or (3) review and send to the next stop and document any concerns. The Task Force lead then reviews the request and, in some cases, may take the request to the DGTF for further discussion. Lastly, based on the recommendations, the Task Force lead makes a determination from the following: (4) approves external sharing with the Data Use Agreement through Sponsored Programs, (5) approves internal sharing with the Internal Data Use Agreement, or (6) confirms redirection of the researcher to existing resources within Health Care.

### Metrics

An analysis of our external data sharing requests that occurred during the pilot revealed that 59% of sharing requests go to academic institutions and hospitals, while commercial entities account for 12%. Most of these requests originate from our hospital, health science colleges, or a collaboration between the hospital and health science colleges. Since the initiation of the pilot, this process has been used to support a total 328 requests for external sharing of data since formalization of process in 2021 (Table 1).

**Table 1.** External data sharing requests metrics.

Requests by year	
2021	25
2022	101
2023	119
2024 (July 1)	83
Total	328

Requests by college	
Medicine	230
Public health	18
Multicollege	65
Nursing	5
Dentistry	2
Liberal arts	7
Administration	1
Total	328

Organization sharing	
Academic/Hospital	192
Not specified^a^	58
Commercial^b^	40
Other^c^	16
Professional	8
Federal	8
Foundation	4
Educational Tech	2
Total	328

Data type	
Deidentified	124
Limited	97
Identified	35
Not specified^a^	72
Total	328

*N* subjects in dataset	
*n* < 50	91
*n* = 50-500	121
*n* = 501-4999	61
*n* ≥ 5000	36
Summary stats	6
Not specified^a^	13
Total	328

Consenting subjects	
Yes	101
No	203
Not specified^a^	24
Total	328

a Features that are not specified are features that were either not originally required to be captured but were later added or added as new features for decision-making during the pilot phase.

b Clinical trials technology, data, data analytics, device, diagnostics & testing, medical technology, consulting, pharmaceutical.

c Community organization, credit reporting agency, nonprofit organization, patient safety organization, public health organization, quality collaborative, research collaborative.

**Figure 3.**
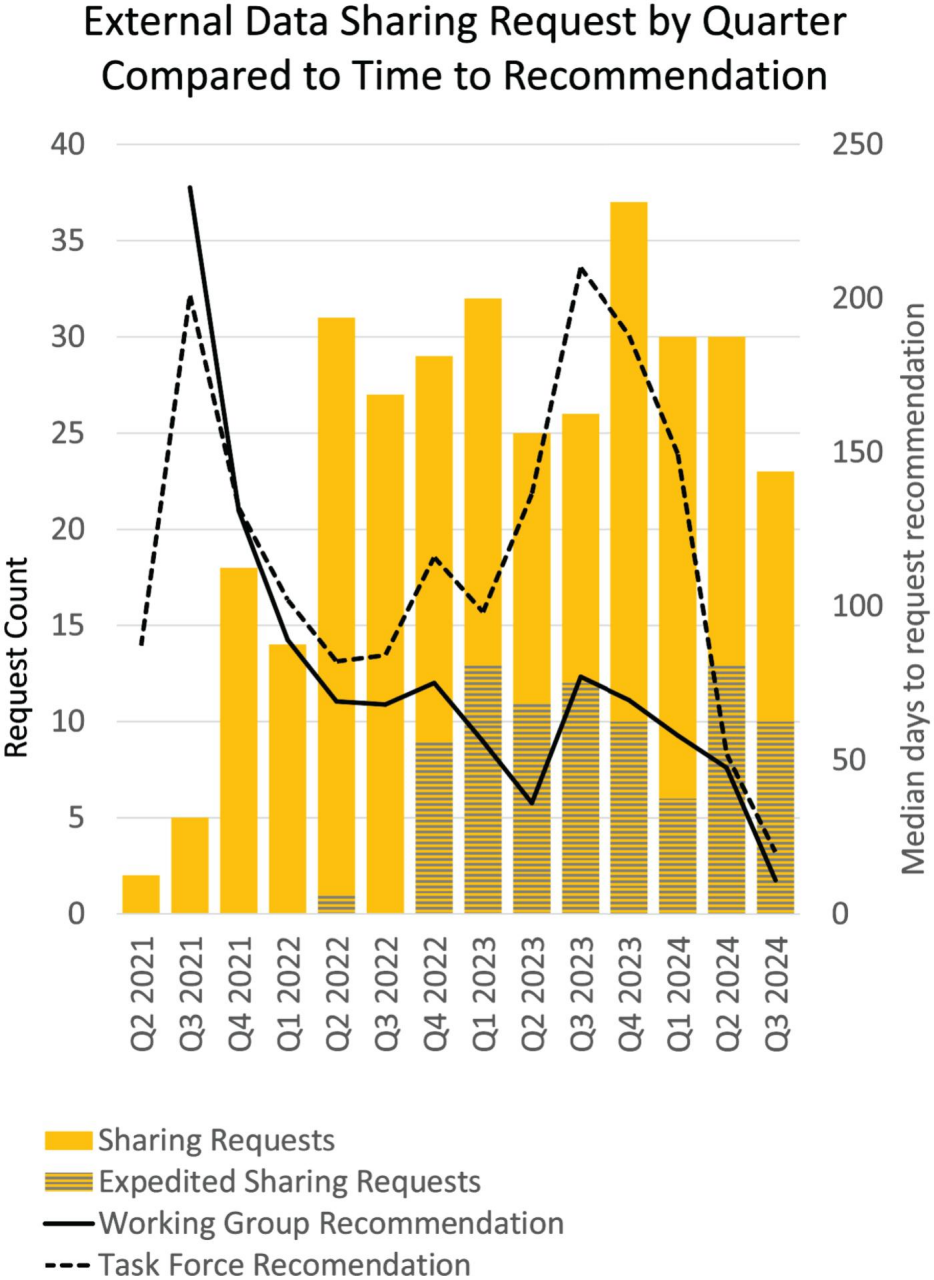
Time to recommendation by the Working Group and Task Force.

The establishment of these processes has also provided the organization with robust tracking of data being shared externally for research purposes and metrics (Figure 3) to guide continuous process improvement.^11^

## Discussion

This paper describes the evolution of a data governance process at an academic health center for sharing real-world data outside the organization and lessons learned from this change. Successfully implementing data governance requires elevation of data literacy throughout the organization, and the impact is measured in the number of studies enabled from this project. Development of these processes established the groundwork for critical decision points such as security reviews for new technologies and practices to address emerging AI technologies. The project was informed by data governance approaches at peer institutions and practices at national networks such as National Covid Cohort Collaborative (N3C). The approach needed to fit the integrated nature of our organization—an academic medical center that is part of a large R1 public university—and a collaborative culture. The external data sharing team’s membership includes representatives from multiple offices, each of which brings specific knowledge critical to optimizing how we use our data for research.

The pilot aimed to test edge cases against the developing models and establish guidelines and processes for handling them. In addition to creating a workflow and process for new data sharing requests, our process also captures existing projects undergoing protocol modifications that may require a data sharing request. When modifications are made to previously reviewed requests involving an Honest Broker, Institutional Review Board (IRB), Data Use Agreement (DUA), or Data Transfer Agreement (DTA), the process owner reviews and recommends whether the modification should be brought back to the WG for discussion on its impact. Fundamental changes, such as new groups not specified in the contract or different phenotypes or data features, may require a new review.

Formalizing the process reduced the bottleneck that existed, enabled data sharing that was not possible previously and improved access to data overall. Tracking metrics during the pilot phase as the landscape moved from disparate processes, with segregated communication over multiple offices to a group charged with a single process and tool to review key elements from the proposed data sharing provided regular landmarks. It exposed gaps in information needed to make informed recommendations, identified the opportunity to create an expedited review for participants consented for data sharing and let the Working Group set target milestone for expected timing of the reviews. As the pilot progressed, the Working Group and Navigator experienced an unintended consequence of the new processes and workflows in that researchers and groups with adjacent review processes would push their review through the External Data Sharing Review Request. This initially resulted in a second bottleneck as these reviews needed to be reviewed by the Working Group and the Task Force, triaged to other offices and communication provided the researchers the pathway for those reviews. However, this also revealed that the process was working effectively in identifying existing projects requiring review.

Establishing information to be documented and metrics from inception has allowed for better process management, measurement, and allowed the group locally to now use data-driven insights for continuous quality improvement.^11^ Importantly, documentation clearly indicates sharing was done with institutional review. Future continuous process improvement could involve additional process measurements and qualitative feedback from the research community and Working Group offices to address bottlenecks and opportunities for improvement.

### Limitations

A limitation of this approach is that it requires review from a minimum of 5 offices which can cause delays when corresponding documentation is incongruent (eg, information in the sharing request does not match the information in the IRB protocol). The process may be perceived as cumbersome, especially for researchers navigating this process for the first time. Community outside of HCE had differing views on whether this process is necessary, particularly beyond the scope of the IRB approval process. In this, there was an opportunity to raise awareness and literacy around data governance and sharing data external to health care. Although each office strives to inform the research community of various requirements, researchers are ultimately responsible for understanding and following the required practices. Additionally, while this process provides a single system for governance and review, with multiple levels of oversight and systems connected with complex research needs, researcher awareness, and adherence are a challenging gap to overcome.

While some aspects of this approach are institution specific, key components are readily generalizable: (1) awareness and understanding: ensure that data decision makers, including senior leaders (eg, Chief Medical Officer (CMO) or Chief Research Officer (CRO)), fully comprehend the data sharing process. (2) Compliance checkpoints: identify and adhere to necessary compliance checkpoints from various perspectives, including human subjects, privacy, legal, security, and contracting. (3) Data brokerage: define data brokerage and practices to ensure data sharing is done correctly and ethically. (4) Expert representation: involve experts who understand the data domain to guide the sharing process effectively. (5) Clear documentation: document the shared data comprehensively to facilitate understanding and future use. (6) Process definition: clearly define acceptable processes for data sharing within the organization.

## Conclusion

This effort collaboratively formalized workflows and detailed documentation processes that allows the Working Group and Task Force to track metrics. Establishing metrics and measurable outcomes from the inception has created improved understanding of data being shared, more efficient process management, measurement, and continuous quality improvement. As innovative and disruptive technologies arise, workflows, policies, and practices need to continue to be reviewed and confirmed or revised. As with most efforts involving health-care data, there continues to be a need for better data literacy.

## Supplementary Material

ooaf041_Supplementary_Data

## Data Availability

The data and materials underlying this article will be shared upon reasonable request to the corresponding author.
