# Supplementary material for: Establishing data governance for sharing and access to real-world data: a case study
Source: JAMIA Open. 2025 Jun 23;8(3):ooaf041. doi: 10.1093/jamiaopen/ooaf041 (PMC12206003; doi:10.1093/jamiaopen/ooaf041)
Supplement: ooaf041_Supplementary_Data [file ooaf041_supplementary_data.zip › Appendix A.docx]

**APPENDIX A**

**Key Data Elements collected for External Data Sharing Requests (*modified from original, **added during iterative process)**

1. Request ID**
2. Principal Investigator Name
3. Principal Investigator Department /Unit**
4. Principal Investigator College/Org**
5. Principal Investigator Title**
6. Principal Investigator Email**
7. Study / Project Title
8. IRB#
9. Corresponding Data Request ID**
10. Date Submitted
11. Service Contact Name** (e.g., person that asked you to complete the External Data Sharing request e.g., Jayne Smith - Human Subjects Office)
12. Service Contact Email**
13. Person Submitting Request**
14. Person Submitting Request Email**
15. Will data be shared outside the Health Care environment? (Yes | No)**
16. What is the estimated number of records to be transferred?*
17. Are all subjects consented to this study? (Yes | No)**
18. If so, does the informed consent include a description of the planned use of the data? (Yes | No)**
19. Name of the third-party entity:
20. How is the data being shared? (e.g., Secure File Transfer Protocol (SFTP))*
21. Will the data be pushed or pulled? (Pushed | Pulled)*
22. How often will you be transferring data? (One Time | Recurring | Other Interval)*
23. Describe other interval*
24. Length of Engagement: (# of months this data will be stored outside of the healthcare covered entity.)
25. Will there be secondary sharing/linking to additional entity? (Yes | No)*
26. If yes, describe secondary sharing/linking to additional entity:
27. What is the purpose of data sharing? (national registry, development partnership, financial incentives, intellectual property development/research, etc.)?
28. What will the data be used for? (Research | QA/QI | Other)*
29. Data Tier (Tier 1 | Tier 2 | Tier 3 | Tier 4)**
30. Will the data elements contain PHI or other sensitive data? (Yes | No)*
31. Type of Data that will be sent? Select All (Patient | Student | Financial | Employee | Other)*
32. Describe Other Data Type
33. Source of the data being sent? Select All (Healthcare EHR | College of Medicine | Human Resources | Student Information | EDW4R (CTSA, UI BioShare, Axiom, Tumor Registries, REDCap)| Other Data Source)*
34. Describe other data source: additional]
35. Which HIPAA data elements does this request contain? Select All (Name | Address | Dates | Telephone numbers | Fax number | Email address | Social Security Number | Medical record number | Health plan beneficiary number | Account number | Certificate or license number | Vehicle identifiers and serial numbers, including license plate numbers | Device identifiers and serial numbers | Web URL | Internet Protocol (IP) Address | Finger or voice print | Photographic image - Photographic images are not limited to images of the face. | Any other characteristic that could uniquely identify the individual, including but not limited to numbers or codes. | This request does not contain any HIPAA identifiers)**
36. Type of Data: (De-Identified Data Set | Limited Data Set | Identified Data Set)**
37. List of data elements: (please provide complete list of data elements)
38. Who is creating the data extract? ((Trusted Broker) - What team is creating this data set)*
39. Where will the data be stored? (University Healthcare secure server | University secure server | Other Non-enterprise storage | External to organization (Other organization) | Other)*
40. Other Data Storage
41. Describe the data access controls of the system you are requesting to move data to?
42. Who will be able to see the data?
43. What is your data management plan?**
44. What happens to data after engagement?
45. Is there a need for a backup of the files being sent (for data provenance)? (Yes | No)*
46. If this data is shared to access computational resources, please describe the computational algorithms or techniques to be used.
47. If intellectual property is developed, who will own it?
48. Any other relevant information that you would like to share?
