# Supplementary material for: Establishing data governance for sharing and access to real-world data: a case study
Source: JAMIA Open. 2025 Jun 23;8(3):ooaf041. doi: 10.1093/jamiaopen/ooaf041 (PMC12206003; doi:10.1093/jamiaopen/ooaf041)
Supplement: ooaf041_Supplementary_Data [file ooaf041_supplementary_data.zip › Appendix B.docx]

**Appendix B**

**Supplemental Material External Data Sharing Recommender**

<https://redcap.link/NeedGovernanceExample>
